# Adipose Tissue Remodeling: Its Role in Energy Metabolism and Metabolic Disorders

**DOI:** 10.3389/fendo.2016.00030

**Published:** 2016-04-13

**Authors:** Sung Sik Choe, Jin Young Huh, In Jae Hwang, Jong In Kim, Jae Bum Kim

**Affiliations:** ^1^Department of Biological Sciences, National Creative Research Initiatives Center for Adipose Tissue Remodeling, Institute of Molecular Biology and Genetics, Seoul National University, Seoul, South Korea

**Keywords:** adipose tissue, adipose tissue macrophage, hypertrophic adipocyte, inflammatory response, iNKT cell, metabolic disorder, obesity

## Abstract

The adipose tissue is a central metabolic organ in the regulation of whole-body energy homeostasis. The white adipose tissue functions as a key energy reservoir for other organs, whereas the brown adipose tissue accumulates lipids for cold-induced adaptive thermogenesis. Adipose tissues secrete various hormones, cytokines, and metabolites (termed as adipokines) that control systemic energy balance by regulating appetitive signals from the central nerve system as well as metabolic activity in peripheral tissues. In response to changes in the nutritional status, the adipose tissue undergoes dynamic remodeling, including quantitative and qualitative alterations in adipose tissue-resident cells. A growing body of evidence indicates that adipose tissue remodeling in obesity is closely associated with adipose tissue function. Changes in the number and size of the adipocytes affect the microenvironment of expanded fat tissues, accompanied by alterations in adipokine secretion, adipocyte death, local hypoxia, and fatty acid fluxes. Concurrently, stromal vascular cells in the adipose tissue, including immune cells, are involved in numerous adaptive processes, such as dead adipocyte clearance, adipogenesis, and angiogenesis, all of which are dysregulated in obese adipose tissue remodeling. Chronic overnutrition triggers uncontrolled inflammatory responses, leading to systemic low-grade inflammation and metabolic disorders, such as insulin resistance. This review will discuss current mechanistic understandings of adipose tissue remodeling processes in adaptive energy homeostasis and pathological remodeling of adipose tissue in connection with immune response.

## Introduction

The adipose tissue is a critical regulator of systemic energy homeostasis by acting as a caloric reservoir. In excess nutrient conditions, the adipose tissue stores surplus nutrients in the form of neutral lipids, whereas in nutrient deficit conditions, it supplies nutrients to other tissues through lipolysis (1). In the past several decades, overnutrition and reduced daily activity have greatly increased obesity rates worldwide, creating a global health emergency due to concomitant increases in insulin resistance, type 2 diabetes, heart disease, atherosclerosis, hypertension, and many types of cancer (2–4). In response to alterations in the energy status, the adipose tissue is rapidly and dynamically remodeled through changes in the number and/or size of adipocytes. Simultaneously, various stromal vascular cells in the adipose tissue undergo numerical and/or functional changes, contributing to the maintenance of the adipose tissue function as an energy reservoir and endocrine organ. This series of events is called “adipose tissue remodeling.” However, under pathophysiological conditions, such as obesity, aberrant adipose tissue remodeling may induce dysregulation of adipose tissue-derived cytokines, hormones, and metabolites, leading to metabolic stresses and disorders in metabolic organs (2–4).

## Functional Complexity of the Adipose Tissue

### The Adipose Tissue as a Specialized Energy Storage Organ

**Figure 1:**
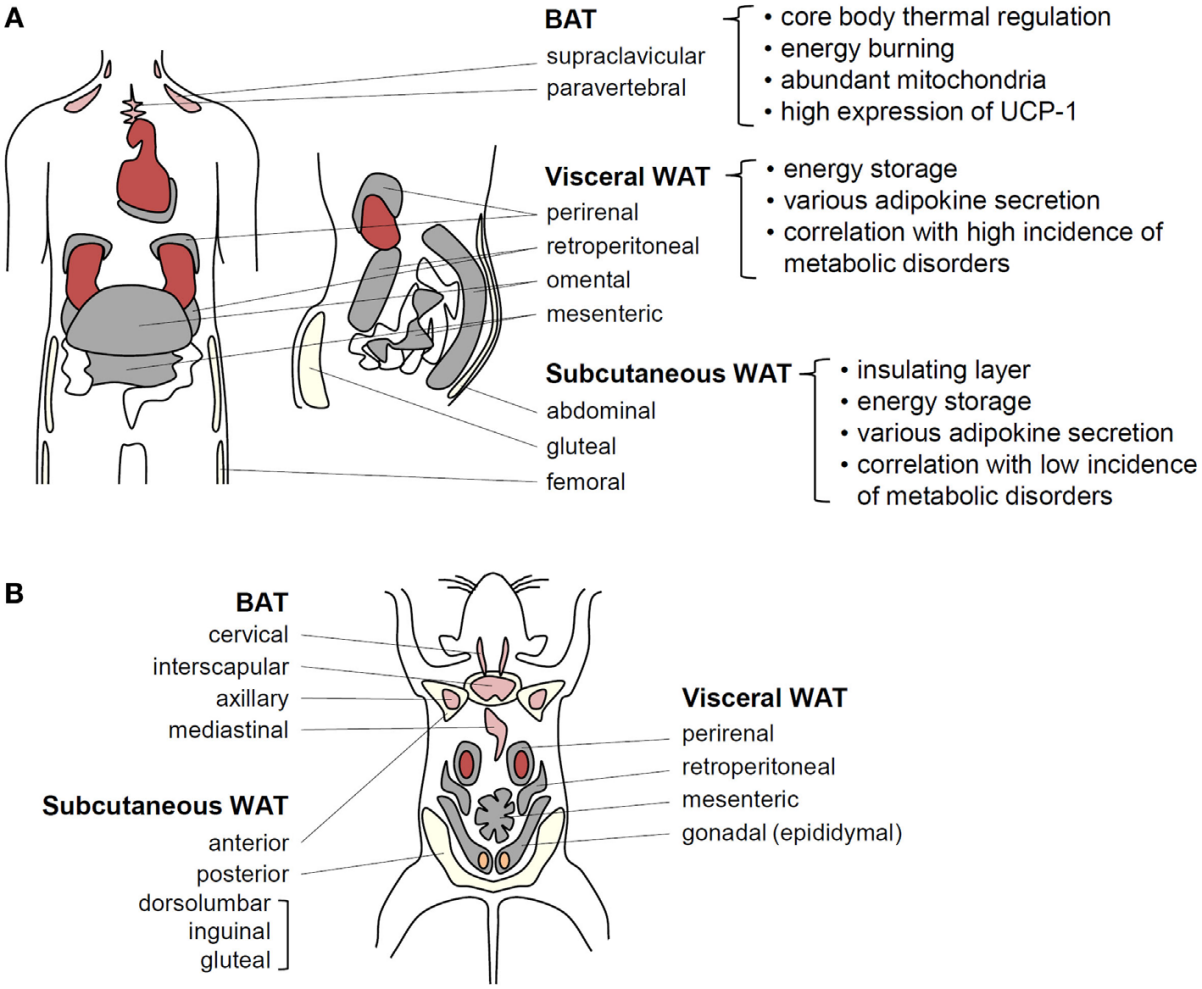
**Adipose tissue functions in energy homeostasis and thermal regulation**. **(A)** In humans, BAT localized around the shoulders and ribs contributes to heat generation. Brown adipocytes exhibit abundant mitochondria and UCP-1 expression related to thermogenesis. It has recently been speculated that BAT efficiency for fat-burning could be harnessed to reduce obesity. Visceral WAT (VAT) and subcutaneous WAT (SAT) possesses considerable capacities for energy storage. VAT surrounds intra-abdominal organs, whereas SAT spreads throughout the body beneath the skin. These fat tissues secrete various adipokines to regulate energy homeostasis. VAT is more strongly associated with obesity-induced metabolic disorders than SAT. **(B)** In adult mice, BAT is well developed and easily observed compared with that in adult humans. Among WAT depots within the abdominal cavity, the paired gonadal depots located around the ovaries in females and the testes in males are studied as a model of VAT. However, these depots do not exist in humans. The paired inguinal depots in the anterior to the upper part of the hind limbs are representative SATs in mice.

Living organisms need to consume energy from their environments to survive. In particular, storage of extra energy obtained during food abundance is an essential physiological activity that enhances survival during food scarcity periods. Multicellular organisms have evolved specialized cells or organs to store excess nutrients as lipids because lipids have higher calories than other nutrients. For example, *Caenorhabditis elegans* stores surplus energy in the form of lipids in intestinal cells (5), whereas sharks and *Drosophila* accumulate excess lipids in the liver and fat body, respectively (6, 7). In other organisms, particularly some fishes and higher vertebrates, the adipose tissue functions as a specialized energy reservoir (1). The adipose tissue is distributed throughout the body and is capable of expanding to accommodate excess energy in the form of accumulated lipids, characteristics distinguishing the adipose tissue from other organs and tissues (8). In humans, there are two major types of adipose tissues, white adipose tissue (WAT) and brown adipose tissue (BAT). Anatomically, WAT comprises two major depots, subcutaneous WAT (SAT) and visceral WAT (VAT) around internal organs. VAT, which is concentrated in the abdominal cavity, is further subdivided into mesenteric, omental, perirenal, and peritoneal depots (Figure 1) (8, 9). The key physiological functions of WAT are insulation and energy storage. In obesity, however, excess VAT is closely linked to metabolic complications, such as insulin resistance and type 2 diabetes (8, 9). Mesenteric and omental adipose tissues are particularly important for hepatic insulin resistance and steatosis because liver is directly exposed to releasing factors from these adipose tissues via the portal vein. As an animal model, rodents have gonadal adipose tissue that is considered part of VAT. However, because of lack of gonadal adipose tissue in humans, careful interpretation is required to extrapolate findings in rodents to human. BAT is a specialized form that participates in non-shivering thermogenesis through lipid oxidation (10, 11). The distinct brown color of BAT is attributed to its high mitochondrial density, which is critical for heat generation and lipid oxidation (10, 11). Although BAT is readily observed in both infant and adult rodents, it has been proposed that BAT in humans is limited to neonates and is gradually replaced by WAT with aging. However, recent positron emission tomography/computed tomography studies have shown that BAT is viable and functional in human adults (12, 13). Thus, various adipose tissues act as central regulators of energy homeostasis by storing excess energy as well as by controlling thermogenesis.

### The Adipose Tissue as an Endocrine Organ Regulating Energy Homeostasis

The adipose tissue has been historically considered merely an energy storage depot, but this concept was revised following the discovery of leptin, the first adipocyte-derived cytokine, by Friedman’s group in 1990. Leptin release in response to changes in nutritional status indicates that the adipose tissue acts as an endocrine organ involved in modulating energy homeostasis (14, 15). Since this discovery, additional cytokines, hormones, and peptides secreted by adipocytes, collectively termed as “adipokines,” have been identified and intensively investigated for roles in the control of energy homeostasis (16). Leptin, one of the most well studied adipokines, is secreted in response to food intake and inhibits appetite by regulating neural circuits located in the brain. Leptin acts on surface receptors expressed in AGRP neurons in the lateral hypothalamus and POMC neurons in the medial hypothalamus to inhibit appetite and stimulate satiety, respectively (17–19). Mice homozygous for the leptin mutation *ob/ob* or leptin receptor mutation *db/db* are massively obese because of uncontrolled appetite and ensuing excess food intake (20, 21). Leptin also promotes lipid oxidation and mitochondrial biogenesis and accelerates energy expenditure in peripheral tissues through both local signaling and regulation of brain-derived factors (22, 23). Circulating leptin is actually elevated in obesity, but hypothalamic leptin resistance aggravates obesity through inhibition of appetite control and lipid oxidation (17–19).

Adiponectin is another adipocyte-secreted adipokine that is abundant in the blood (24–26). Adiponectin exerts anti-obesity and antidiabetic effects and alleviates insulin resistance by stimulating lipid oxidation and anti-inflammatory responses (27–32). There are two major receptors for adiponectin AdipoR1 and AdipoR2. Both receptors stimulate AMP-activated protein kinase (AMPK), which is necessary for the anti-obesity and antidiabetic actions of adiponectin (29–31). There are two distinct forms of circulating plasma adiponectin, a low molecular weight (LMW) full-length trimer and a hexamer. The full-length trimer aggregates via disulfide bonding into high molecular weight (HMW) multimers of 12–18 monomers. It has been reported that these multimers are the primary bioactive forms and that each may exert different biological effects. In obesity, the circulating plasma levels of HMW and LMW adiponectin are reduced, and this altered adiponectin oligomeric profile has been suggested to be a reliable clinical indicator of metabolic disorders (33).

Recent studies have identified additional adipokines that promote obesity and associated morbidities (34–37). For instance, resistin, an adipokine mainly secreted from VAT, is elevated in obesity. In mice, administration of resistin impairs glucose tolerance and insulin action, implying that elevated levels of resistin may link obesity to diabetes (34). Given that the resistin receptor has not been identified, the molecular pathways through which resistin induces insulin resistance in obesity are still uncertain. Retinol-binding protein 4, another adipokine upregulated in serum of insulin-resistance mice and humans, impairs insulin signaling in liver and muscle (35). In contrast, omentin, identified in the human omental adipose depot, regulates blood glucose level by enhancing insulin action and is decreased in obesity (36, 37). The recently identified adipokine nesfatin, primarily derived from SAT, modulates appetite according to the nutrient status (38). In addition to peptide adipokines, lipid metabolites called “lipokines” have also been discovered. In the adipose tissue, C16:1n7-palmitoleate is synthesized *de novo* via stearoyl-CoA desaturase-1. Secreted C16:1n7-palmitoleate improves muscle insulin sensitivity and suppresses fat accumulation in the liver (hepatosteatosis) (39). The identification of multiple adipokines and lipokines with disparate cellular actions underscores the central role of adipose tissue depots as endocrine organs in the dynamic regulation of systemic energy homeostasis.

### The Adipose Tissue as a Thermal Regulator

Compared with WAT, BAT is characterized by multilocular lipid droplets, rich vascularization, and abundant mitochondria. Unlike WAT, BAT functions prominently in thermoregulation through lipid oxidation-mediated heat generation (10, 11). Although both BAT and muscles harbor high levels of mitochondria, BAT is specialized for heat generation rather than for ATP synthesis by high expression of mitochondrial uncoupling protein 1 (UCP-1) (10, 11). The adipose tissues express high levels of β-adrenergic receptors that mediate cold-induced lipolysis (40). After cold exposure, it appears that large amounts of lipids from WAT flow into BAT (40). Concurrently, β-adrenergic signaling in BAT activates the expression of peroxisome proliferator-activated receptor γ coactivator 1α (PGC-1α), which stimulates the expression of UCP-1 and mitochondrial genes (41). In addition to UCP-1 expression, cold-induced BAT enhances lipid uptake for the efficient production of heat, accompanied by mitochondrial biogenesis (40). In accordance with this general model of homeostatic thermogenesis, it has been reported that cold exposure elevates the expression of pro-angiogenic factors, including vascular endothelial growth factor (VEGF), and impedes the expression of anti-angiogenic factors in BAT (42). VEGF receptor (VEGFR) 2 blockage abolishes cold-induced angiogenesis and impairs thermogenic capacity, indicating that angiogenic remodeling of BAT is one of important factors for thermoregulation (42).

Emerging evidence suggests that both BAT and SAT contribute to thermal regulation (43). Cold exposure induces “beiging” remodeling of SAT to the BAT phenotype (referred to as “adipose tissue browning”) (40, 43). Although beige/bright adipocytes and brown adipocytes share the ability for thermogenesis, there are several differences. First, myogenic factor five-negative beige adipocytes are derived from different developmental lineages compared with myogenic factor five-positive brown adipocytes (44). Second, even though PRD1-BF-RIZ1 homologous domain-containing protein-16 (PRDM16) has been recognized as a critical transcriptional coactivator for both brown and beige adipocytes, PRDM16 depletion impedes beige adipocyte development upon β-adrenergic stimulation but has no effect on brown adipocyte development or function (45, 46). To date, two hypotheses have been proposed for beige adipocyte differentiation: one is that beige adipocytes are derived by transdifferentiation of mature white adipocytes and the other is that beige adipocytes are differentiated from specific precursors (47–51). Permanent or inducible labeling of adipocyte lineages in mice demonstrated that the increased number of subcutaneous beige adipocytes after cold exposure resulted from *de novo* adipogenesis (51), indicating that there are distinct precursor cells capable of differentiating into beige adipocytes in SAT. It has also been reported that temperature can reverse this transformation in SAT. Beige adipocytes formed by cold stimulation revert to typical white adipocytes after warm adaptation (50). Moreover, subsequent cold stimulation reconverts these white-typical adipocytes to beige adipocytes. These results suggest the potential for repeated transdifferentiation of white adipocytes into beige adipocytes. Although it remains controversial whether mouse beige adipocytes have an equivalent cell population in human SAT, it has been demonstrated that human adult BAT consists of both classical brown adipocytes and beige adipocytes (52, 53). Moreover, recent marker gene analyses have suggested that the supraclavicular BAT of adult humans appears to consist of beige adipocytes rather than classical brown adipocytes (52–55).

Several cytokines are involved in the stimulation of beiging and/or browning. For instance, fibroblast growth factor 21 (FGF21) activates PGC-1α and accelerates the function of brown adipocytes (56–58). The newly identified adipokine irisin has been reported to link muscle-induced shivering thermogenesis to FGF21-induced brown (beige) adipose tissue-mediated non-shivering thermogenesis (59). Bone morphogenetic proteins (BMPs) 4 and 7 are also associated with the browning of adipocytes (60, 61). A better understanding of BAT activation and WAT browning may lead to the discovery of valuable therapeutic targets for the treatment of obesity and metabolic diseases (40, 43, 62). Despite these findings, more studies are needed to elucidate the molecular mechanisms of beiging/browning of adipocytes as well as their developmental origins.

## Hypertrophic and Hyperplasic Adipose Tissue Expansion

### Two Modes of Adipose Tissue Expansion

**Figure 2:**
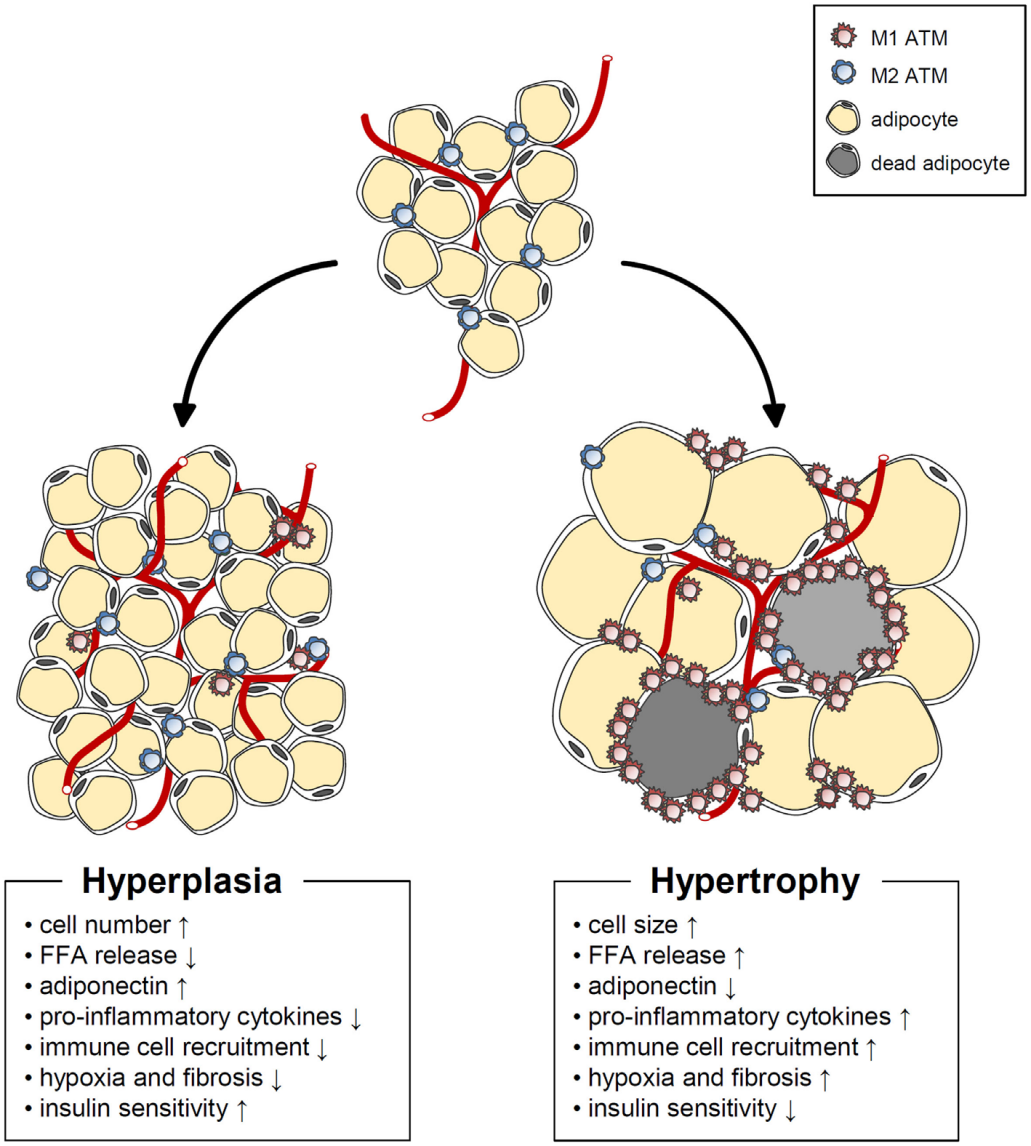
**Characteristics of hypertrophic and hyperplasic adipocytes**. In obesity, adipose tissue expansion occurs by two different mechanisms. Hypertrophic adipose expansion through increased adipocyte size is associated with such harmful phenomena as increased basal fatty acids release, pro-inflammatory cytokine release, immune cell recruitment, hypoxia, fibrosis, decreased adiponectin, and impaired insulin sensitivity. On the other hand, hyperplasic adipose expansion though increased adipocyte number is linked to beneficial phenomena, such as increased adiponectin, decreased basal fatty acids release, pro-inflammatory cytokine release, immune cell recruitment, hypoxia, fibrosis, and improved insulin sensitivity.

Adipose tissue expansion in obesity could be mediated by hypertrophy (enlarged adipocytes), hyperplasia (increased numbers of adipocytes), or both (Figure 2). Adipocyte hypertrophy and hyperplasia are regulated by environmental and genetic factors (63). However, it is still uncertain how these two modes of adipose tissue expansion are controlled at the molecular level. In humans, SAT develops during weeks 14–24 of fetal gestation from the head and neck to the trunk and limbs through an increase in the number of adipocytes. By 28 weeks, major fat depots are organized (64). During the first year after birth, both adipocyte number and size increase in humans. The number of adipocytes then appears to remain stable until adolescence, when it increases once again (65, 66). In contrast to SAT development, very little is known about the developmental period of VAT. It has been shown that VAT is rarely formed before birth and that the total amount of VAT remains small until adolescence (67, 68).

Both SAT and VAT masses are markedly increased in obese humans. In childhood obesity, WAT mass expansion is accompanied by adipocyte hyperplasia (69, 70). In obese animal models, it has been proposed that *de novo* adipogenesis begins when the adipocytes reach an upper volume limit (51, 71). Thereafter, formation of new adipocytes from precursor cells is required for further increases in energy storage capacity. In adult humans, however, the increase in WAT mass that triggers metabolic disorders is primarily due to adipocyte hypertrophy (72, 73). Spalding et al. reported that adipocyte numbers are roughly constant in lean and obese adults (74). In addition, neither the number nor the turnover rate of adipocytes during adulthood is altered by obesity or weight loss, implying that adipocyte hypertrophy is the predominant contributor to adult obesity.

### Hypertrophic Adipocytes

**Figure 3:**
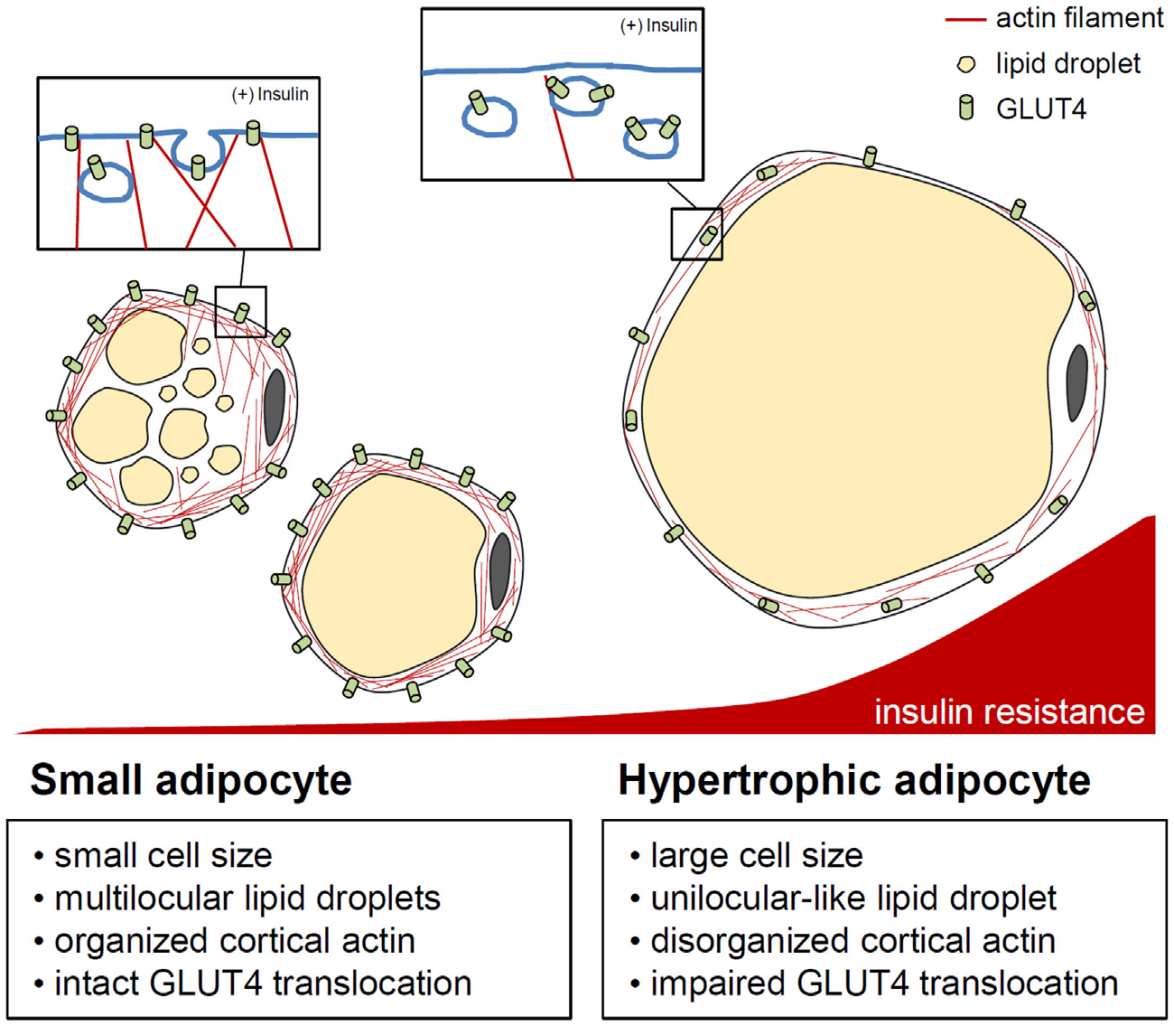
**Actin cytoskeleton and insulin-stimulated GLUT4 translocation control in adipocytes**. In adipocytes, cytosolic and cortical actin organization is involved in GLUT4 storing vesicle (GSV) transport by insulin stimulation. When adipocytes are hypertrophied, enlarged unilocular lipid droplets and expanded cell volume may impede cortical actin dynamics, resulting in improper/deficient translocation of GSVs. This indicates the importance of the adipocyte cytoskeleton in the regulation of adipocyte glucose metabolism in response to insulin.

In recent decades, many studies have suggested that adipocyte hypertrophy *per se* results in abnormal adipocyte function and leads to insulin resistance (75–79). The distinguishing features of hypertrophic adipocytes are as follows. First, in the obese adipose tissue, hypertrophic adipocytes show necrotic-like abnormalities (80–82). Necrosis-like adipocyte death is increased in hormone-sensitive lipase-deficient mice with adipocyte hypertrophy as well as in diet-induced obesity (DIO) mouse models (80–82). It has been proposed that an increase in dead adipocytes in obesity will impede adipose tissue function and induce inflammation. Second, hypertrophic adipocytes exhibit increased expression and secretion of pro-inflammatory cytokines, including tumor necrosis factor α (TNFα), interleukin (IL)-6, IL-8, and monocyte chemoattractant protein-1 (MCP-1) (83). This elevation of pro-inflammatory cytokines leads to serine phosphorylation of insulin receptor substrate-1 via nuclear factor κB and Jun N-terminal kinase signaling, resulting in the development of insulin resistance (84, 85). Pro-inflammatory cytokines also promote adipose tissue inflammation by recruiting various immune cells, including macrophages and T cells (2–4). Third, adipocyte hypertrophy induces local adipose tissue hypoxia because of a relative deficiency of vasculature (86–88). When hypoxia occurs in the adipose tissue, the expression levels of angiogenic factors and inflammatory response-associated genes are upregulated (86, 88, 89). Activation of hypoxia-inducible factor (HIF) 1α, a key transcription factor mediating hypoxic responses, accelerates adipose tissue fibrosis. Also, HIF1α augments the local inflammatory response in the obese adipose tissue, whereas HIF2α attenuates adipose tissue inflammation in obesity (87, 90, 91). Moreover, basal lipolysis is elevated in hypertrophic adipocytes (92, 93), increasing the leakage of free fatty acids (FFAs). Large amounts of FFAs released from the obese adipose tissue are taken up by other tissues, such as the liver and muscle, which can cause ectopic lipid accumulation and lipotoxicity (94, 95). Furthermore, saturated fatty acids, such as palmitic or stearic acids, activate the Toll-like receptor 4 signaling cascade, which plays an essential role in innate immunity, leading to chronic inflammation as well as insulin resistance (96–98). It was recently reported that adipocyte hypertrophy also impairs insulin-dependent glucose uptake because of a defect in GLUT4 trafficking (79). Upon chronic treatment with FFAs, 3T3-L1 adipocytes become enlarged with unilocular lipid droplets, mimicking *in vivo* hypertrophic adipocytes. These unilocular and hypertrophic adipocytes show disrupted cortical actin structures and impaired insulin-dependent GLUT4 trafficking to the plasma membrane (Figure 3). Thus, it is likely that adipocyte hypertrophy impairs adipocyte function though both inflammation-dependent and inflammation-independent mechanisms, thereby exacerbating insulin resistance and disrupting energy metabolism.

### Hyperplastic Adipocytes

Adipocyte hypertrophy occurs more readily in SAT than VAT until adolescence (65, 66, 71, 99). For hyperplasic expansion of the adipose tissue, adipocyte precursor cells must differentiate into adipocytes, a process regulated by various transcription factors and hormones. Peroxisome proliferator-activated receptor γ (PPARγ), a key regulator of adipogenesis, and CCAAT/enhancer binding protein families regulate the induction and maintenance of adipogenesis (100–102). Sterol regulatory element-binding transcription factor 1c (SREBP1c) is another key transcription factor that stimulates the expression of lipogenic genes, including acetyl-CoA carboxylase, fatty acid synthase, and saturated fatty acid dehydrogenase. It has been reported that activation of SREBP1c provides endogenous PPARγ ligands and consequently increases adipogenesis (103, 104). Other extracellular signals, including BMPs, insulin-like growth factors, and WNT, modulate adipocyte differentiation (105–108). However, the molecular mechanisms of *in vivo* adipogenesis in normal and obese conditions remain elusive. Recently, several *ex vivo* and *in vivo* studies have identified adipocyte precursor cell types (109–113). In human WAT, adipocyte precursor cells are abundant among CD31^−^ and CD34^+^ stromal vascular fractions (109). In mice, more specific subpopulation of adipogenic precursor cells have been characterized, including highly pro-adipogenic subpopulations of CD45^–^, CD31^–^, Ter119^–^, CD29^+^, CD34^+^, Sca-1^+^, and CD24^+^ cells (110). Subsequent studies have shown that these subpopulations can form fully functional WAT deposits when transplanted into A/Zip lipodystrophic mice, implying that these adipocyte precursor cells can proliferate and differentiate into adipocytes *in vivo*. However, it remains unknown how and when adipocyte precursor cells can commit to adipocyte differentiation.

Many cohort studies have reported that several common parameters of obesity, including body mass index (BMI), insulin resistance, and cardiovascular diseases, are not always positively correlated with obesity. For instance, certain obese individuals with BMIs over 30 kg/m^2^ are insulin sensitive and exhibit normal metabolic parameters, including blood glucose and lipid levels, HOMA-IR, and plasma inflammatory markers. Such obese individuals are called “metabolically healthy obese” (MHO) (77, 114–117). A frequent feature of the MHO population is a lower number of immune cells and a higher proportion of relatively small adipocytes in adipose tissues (77, 114, 115, 117). Moreover, there are several mouse models that show similar MHO phenotypes (118, 119). Thiazolidinedione derivatives like rosiglitazone are antidiabetic agents that promote adipogenesis by acting as PPARγ ligands (120, 121). *De novo* small adipocytes are detected in obese *db/db* mice treated with rosiglitazone, and insulin-dependent glucose uptake is enhanced in rosiglitazone-induced newly differentiated small adipocytes (79, 122). These findings suggest that *de novo* differentiation of small adipocyte could ameliorate insulin resistance in obesity by providing additional capacity to store excess energy. Although the implications of adipocyte hyperplasia in the adipose tissue function are not fully understood, the regulation of hyperplasic adipocytes may exert beneficial effects against adipocyte hypertrophy and subsequent insulin resistance.

## The Adipose Tissue Inflammation and Metabolic Complications

### Obesity and Adipose Tissue Inflammation

Inflammation is a biological defense response against harmful stimuli, such as pathogen invasion and cell damage (123). A close relationship between the inflammatory response and insulin resistance has been proposed on the basis of the suppression of insulin-dependent glucose uptake in sepsis patients (124, 125). Furthermore, the anti-inflammatory drug salicylate alleviates insulin resistance in diabetic subjects (126, 127). Expression of the inflammatory cytokine TNFα is increased in the obese adipose tissue, whereas blockade of TNFα/TNFα receptor signaling by TNFα neutralization improves insulin-dependent glucose uptake (128). Other pro-inflammatory cytokines, such as IL-1β, IL-6, and MCP-1, are also upregulated in the obese adipose tissue (129). These results indicate that inflammatory cytokines secreted from the adipose tissue contribute to the induction of insulin resistance.

**Figure 4:**
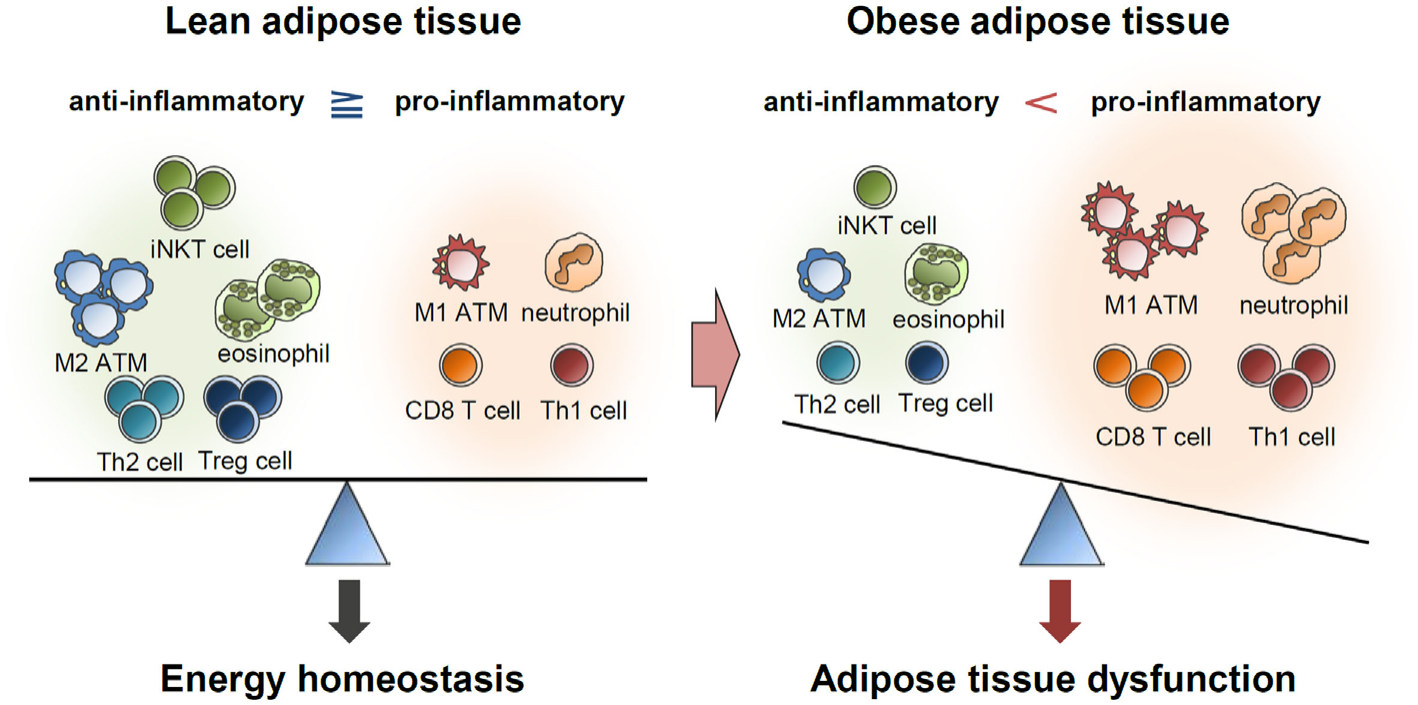
**Balance of immune responses in the regulation of adipose tissue function**. Lean adipose tissue harbors various anti-inflammatory immune cells, such as eosinophils, M2 macrophages, Th2 cells, iNKT cells, and Treg cells. These immune cells help in maintaining insulin sensitivity and store extra energy in the form of TGs. In obese adipose tissue, the numbers of pro-inflammatory immune cells, including neutrophils, M1 macrophages, mast cells, Th1 cells, and CD8 T cells, are greatly elevated. Simultaneously reduced number of anti-inflammatory immune cells accelerates pro-inflammatory response and adipose tissue dysfunction.

Immune cells residing in the adipose tissue actively secrete numerous pro- and anti-inflammatory cytokines (2–4). Anti-inflammatory cytokines help in maintaining insulin sensitivity in the lean adipose tissue, while accumulation of pro-inflammatory cytokines in obesity leads to insulin resistance (2–4) (Figure 4). In addition, pro-inflammatory cytokines stimulate lipolysis in adipocytes, leading to lipotoxicity in other tissues (2, 3). In *C57BL/6* mice, an inflammatory response was specifically induced in the adipose tissue but not in other metabolic tissues by a brief (1 week) high-fat diet (HFD) (130). During long-term HFD, however, pro-inflammatory responses were also greatly enhanced in other metabolic tissues, including the liver and muscles (130). This report proposes that inflammatory responses because of excess energy intake are primarily initiated in adipose tissues and that chronic inflammation of adipose tissues subsequently induces inflammation in other metabolic organs, such as the liver, muscle, and pancreas. Therefore, it is likely that inflammation is a causal factor for widespread systemic insulin resistance in obesity.

### Pro-inflammatory Cytokine-Producing Immune Cells

Adipose tissue remodeling is accompanied by reversible changes in immune cell composition as well as adipocyte size, altering numerous adipose tissue functions (2–4). The numbers of neutrophils and macrophages are rapidly increased in the adipose tissue upon HFD feeding (130–132). Neutrophils are the most abundant blood leukocyte type and rapidly react to inflammatory responses (133). It was reported that the expression levels of neutrophil-derived elastase and myeloperoxidase (MPO) were elevated after only 3 days of HFD feeding in mice and that neutrophils quickly accumulated in the obese adipose tissue (131, 132). Both HFD-induced adipose tissue inflammation and insulin resistance were attenuated in mice treated with a neutrophil elastase inhibitor and in neutrophil elastase knockout mice (132), implying that neutrophil elastase contributes to the pro-inflammatory response and insulin resistance in adipose tissues. However, one study reported that MPO-positive cells did not infiltration into adipose tissues of obese humans even though the levels of circulating MPO and neutrophils were elevated (134).

Macrophages are the most abundant cell type among adipose tissue leukocytes, constituting almost 50% of total immune cells (135, 136). The number of macrophages in adipose tissue prominently increases with obesity. Accumulated macrophages are frequently observed in a crown-like structure (CLS) surrounding lipid droplet-associated protein (perilipin)-negative adipocytes in obese subjects (80, 81, 137). MCP-1 is a well-known chemokine that attracts macrophages into the adipose tissue in obesity. Both MCP-1-deficient mice and MCP-1 receptor CCR2-deficient mice are protected against macrophage accumulation and inflammatory responses in adipose tissues and are resistant to DIO-induced insulin resistance (138, 139). Adipose tissue macrophages (ATMs) can be divided into M1 (classically activated) and M2 types (alternatively activated) (2, 140). The M1 type prominently express inducible nitric oxide synthase (iNOS), TNFα, IL-1β, and CD11c on the cell surface, whereas M2 macrophages express arginase, IL-10, and Ym-1. It has been reported that M1 ATMs are the major contributors to adipose tissue inflammation and insulin resistance in obesity. Furthermore, mice transplanted with bone marrow depleted of CD11c-positive cells show attenuated adipose tissue inflammation and insulin resistance during DIO (141). Although this simple dichotomous classification of ATMs has some experimental benefits, it appears that ATMs are composed of diverse cell populations not easily classified as M1 or M2.

The polarization of M1 macrophages can be induced by IFNγ or lipopolysaccharides (142). In the adipose tissue, IFNγ is predominantly expressed in T cells, particularly Th1 and CD8 T cells (142). The numbers of Th1 and CD8 T cells are elevated in adipose tissues of obese subjects (142–144). In mice, CD8 T cell depletion reduces M1 macrophage accumulation and TNFα expression in the adipose tissue and also improves insulin sensitivity (143). Coculture of CD8 T cells and adipose tissue from HFD-fed mice increases CD8 T cells proliferation and TNFα expression (143). These findings suggest that factors induced in the adipose tissue by HFD further activate CD8 T cells. The Th1 cell, a subtype of CD4 T cell, is also a major IFNγ-expressing cell that accumulates in obesity (144). IFNγ stimulates the expression of chemokines and pro-inflammatory cytokines in adipocytes as well as the M1 polarization of macrophages. Although HFD-fed IFNγ knockout mice show no difference in adipose tissue mass, adipose tissue inflammation, and glucose tolerance are ameliorated compared to wild-type mice (142). These findings suggest that pro-inflammatory immune cells amplify the inflammatory response via cell-to-cell interactions with adipocytes and other immune cells, which in turn induces chronic inflammation, adipose tissue dysfunction, and insulin resistance.

### Anti-inflammatory Cytokine-Producing Cells

Other immune cell populations also protect against adipose tissue inflammatory responses. For instance, M2 macrophages strongly express arginase, which sequesters arginine from iNOS, resulting in inhibition of nitric oxide generation and suppression of M1 macrophage function (2). Moreover, M2 macrophages secrete the anti-inflammatory cytokine IL-10 (2). Treatment of adipocytes with IL-10 alleviates TNFα-induced insulin resistance (145). Activation of STAT6 signaling by Th2-type cytokines, such as IL-4, mediates M2 polarization (2). When IL-4 is administered to DIO mice, adipose tissue mass and inflammatory responses are alleviated, and insulin sensitivity is improved (146). IL-4 is primarily produced by eosinophils in murine adipose tissues (147), and compared with wild-type mice, eosinophil-deficient mice are more glucose-intolerant upon HFD feeding and show diminished numbers of M2 ATMs. In contrast, overexpression of IL-5, an eosinophil chemoattractant, induces glucose tolerance and increases eosinophil numbers in the adipose tissue (147). Thus, it has been proposed that eosinophils in the adipose tissue regulate insulin sensitivity by mediating M2 macrophage polarization.

**Figure 5:**
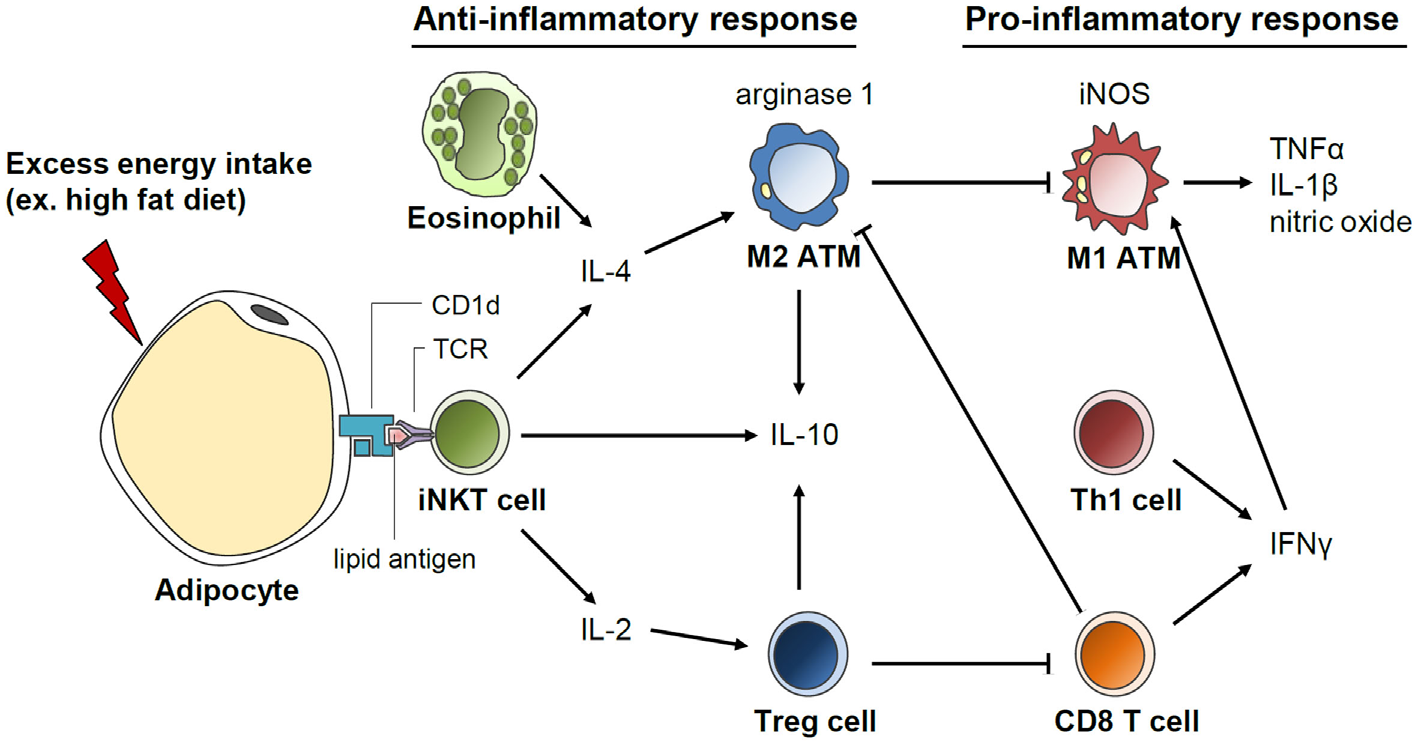
**Invariant natural killer T (iNKT) cell-mediated regulation of anti-inflammatory response in the adipose tissue**. Adipocytes secrete various inflammation-inducing factors including FFAs upon excess energy intake, such as HFD. In addition, antigen presentation by CD1d on adipocytes could activate iNKT cells, which rapidly secrete great quantities of cytokines, such as IL-4, IL-2, and IL-10. IL-4 produced by iNKT cells induces macrophage polarization into M2 type and arginase expression. IL-2 secretion by iNKT cells promotes Treg cell function in the adipose tissue. Activation of anti-inflammatory responses mediated by iNKT cells could play a crucial role in the suppression of excessive pro-inflammatory response in the adipose tissue upon HFD.

Beneficial roles of invariant natural killer T (iNKT) cells in adipose tissue inflammation and insulin sensitivity have recently been identified, which are associated with secretion of anti-inflammatory cytokines, such as IL-4 and IL-10 (Figure 5) (148–152). These iNKT cells have unique functional characteristics. For example, iNKT cells recognize the lipid antigen loaded on CD1d molecules of antigen-presenting cells, whereas CD4 and CD8 T cells recognize the peptide antigens loaded on MHC molecules (153). In addition, iNKT cells can rapidly secrete large quantities of cytokines after activation (153). The number of iNKT cells in adipose tissues is reduced in obese subjects (149, 150, 152). In short-term HFD-fed mice, adipose iNKT cell numbers are rapidly reduced concomitant with enhanced expression of iNKT cell activation markers (152). These results suggest that iNKT cells are involved in the early inflammatory response to metabolic stress in the adipose tissue. In addition, it has been shown that iNKT cell-deficient mice are more susceptible to adipose tissue inflammation and insulin resistance than wild-type mice (150, 152). Moreover, when iNKT cells are supplemented by adoptive transfer or activated by α-galactosylceramide treatment in murine models, body weight gain is reduced; glucose tolerance is improved; and anti-inflammatory cytokines, such as IL-4 and IL-10, are elevated in DIO (149, 150). Under HFD, the expression of the arginase gene is increased in wild type but not in iNKT cell-depleted mice, suggesting a crucial role of iNKT cells in M2 polarization (148). These data suggest that iNKT cells suppress adipose tissue inflammation and insulin resistance in DIO mice. It was recently suggested that iNKT cells in the adipose tissue possess a unique tissue-resident property, unlike iNKT cells in other tissues. Moreover, iNKT cells can regulate the functions of regulatory T (Treg) cells via IL-2 production as well as those of M2 macrophages via IL-10 production in the adipose tissue (154). Further, CD1d molecules that present lipid antigens to iNKT cells are highly expressed in adipocytes that store large amounts of lipid metabolites (151, 152). Despite these recent findings, the roles of adipocyte CD1d under physiological and pathological conditions and the identity of the endogenous lipid antigens exclusively loaded on adipocyte CD1d remain to be elucidated.

Treg cells protect the adipose tissue against severe inflammation through cell-to-cell interactions as well as by the secretion of anti-inflammatory cytokines, such as IL-10 and TGFβ. The number of Treg cells is reduced in adipose tissues of obese humans and mice models (155). In Treg cell-deficient mice, HFD-induced insulin resistance and expression of pro-inflammatory cytokines in the adipose tissue are exacerbated (155). Also, it has been reported that adipose tissue Treg cells have a 136-fold higher level of IL-10 transcripts than that in Treg cells in the lymph nodes (155).

Thus, it appears that diverse anti-inflammatory immune cells, including eosinophils, M2 macrophages, iNKT cells, and Treg cells, can cooperate to resolve inflammatory responses in adipose tissues during the early stages of obesity. Taken together, these results suggest that chronic excessive energy and an imbalanced ratio of pro-inflammatory to anti-inflammatory immune cells induce dysregulated immune responses in obese adipose tissues.

Several studies have also shown that the adipose tissue inflammatory response differs depending on tissue location (81, 137, 156–160). It is well known that VAT expansion is a stronger risk factor for the development of metabolic syndrome and insulin resistance than SAT expansion (70, 161). In addition, obesity-induced macrophage accumulation is greater in VAT than in SAT (137, 156, 158, 159). The mRNA levels of MCP-1, CCR2, and macrophage marker genes, such as CD14 and CD163, are consistently higher in human VAT than in SAT (156, 159). Moreover, IFNγ-secreting CD8 T cells and relative ratios of Th1 and Th2 cells are higher in VAT than in SAT (162). These differences in the inflammatory potential between fat depots need to be further elucidated to understand the numerical changes and roles of each immune cell in obese adipose tissues.

### Dynamicity and Reversibility of Adipose Tissue Immune Cell Balance

In the adipose tissue, the pro-inflammatory response causes insulin resistance, whereas the anti-inflammatory response preserves insulin sensitivity. The degree of inflammation in the adipose tissue is determined by dynamic interactions among various immune cells. For example, in mouse models, HFD feeding enhances M1 polarization to accelerate pro-inflammatory responses in adipose tissues. However, it is noteworthy that absolute number of M2 macrophages in fat tissue weight is also increased by HFD at the early stage of obesity (163, 164). It appears that immune homeostasis is determined by crosstalk between elevated M1 and M2 ATMs despite the change in nutritional status (165). On the other hand, each immune cell population in the adipose tissue exhibits a unique pattern of change under HFD feeding. In mouse models, neutrophils and macrophages are rapidly increased, whereas iNKT cells are decreased in the adipose tissue within 1 week during HFD (130, 132, 152). In contrast, the number of Treg cells remains stable for 10 weeks of HFD feeding (130, 143). These differences suggest that various adipose tissue immune cells have distinct roles in the adipose tissue immune response and may maintain immune balance to prevent uncontrolled pro-inflammatory responses. In short-term HFD-fed mice, changes in immune cell populations are not sufficient to affect glucose tolerance, possibly because of rebalancing of adipose tissue inflammation via crosstalk between local immune cell populations (130). However, under chronic HFD feeding, cumulative effects induced by long-term changes in immune cell populations induce metabolic dysregulation (132, 152). In other words, when obesity is prolonged, the net balance between pro- and anti-inflammation shifts to chronic inflammation. Without sustainable counteraction by anti-inflammatory responses, elevated pro-inflammatory immune cells appear to dysregulate adipose tissue functions with the progress of severe obesity, thereby disrupting glucose and lipid homeostasis.

Recent studies have revealed that adipose tissue remodeling due to inflammation is a reversible phenomenon closely associated with insulin sensitivity and adiposity. In both human obesity and mouse models, expression levels of pro-inflammatory markers and macrophage numbers increase in parallel with insulin resistance. However, after weight loss by calorie restriction, immune responses are re-balanced, and insulin sensitivity is restored by the reduction of M1 macrophages and induction of M2 macrophages (166–168). Furthermore, it has been reported that weight loss in obese and diabetic subjects after bariatric surgery results in reduced inflammatory gene expression and pro-inflammatory immune cell numbers (169–171). Thus, it is likely that rebalancing of pro- and anti-inflammatory immune cells could be driven by external stimuli, such as hormones, cytokines, or chemicals. Activation of M2 ATMs by Th2 cytokines, such as IL-4, attenuates the pro-inflammatory response in inflamed adipose tissues and ameliorates metabolic complications, including insulin resistance (146, 172). These results suggest that immune responses in adipose tissues are reversible and dynamically regulated by factors associated with changes in adiposity and immunostimulation, which eventually restore systemic insulin sensitivity.

## Adipose Tissue Macrophages and Tissue Remodeling

### Adipose Tissue Angiogenesis and Macrophages

In lean subjects, ATMs contribute to the regulation of adipose tissue function as well as adipose tissue remodeling (173). Multiple lines of evidence suggest that ATMs influence angiogenesis in adipose tissues (174–178). In mouse models, ATM depletion through the use of clodronate liposomes reduces blood vessel structures in adipose tissues (178). In addition, macrophages recruited into the tip of the gonadal adipose tissue promote angiogenesis during tissue outgrowth (174). In particular, lymphatic vessel endothelial receptor 1-positive macrophages secrete tissue remodeling factors, such as matrix metalloproteinase (MMP)-7, MMP-9, and MMP-12 that stimulate VEGF/VEGFR signaling for the formation of new vessels (174). It has been suggested that ATMs may be the major cell type that releases MMPs in the human adipose tissue (176). In Matrigel, ATMs isolated from the human adipose tissue promote the formation of endothelial cell tubes by secreting MMPs, such as MMP-9 (176). Extracellular matrix (ECM) remodeling, including the degradation of the connective tissue and basement membrane proteins, is necessary during the early stage of angiogenesis (179, 180). Thus, it has been suggested that ATMs contribute to angiogenic remodeling of the adipose tissue by modulating ECM.

Remodeling of ECM is also associated with the modulation of adipogenesis during adipose tissue expansion. Adipocyte differentiation is regulated by the deposition of collagen, a major protein component of ECM (181). In addition, it has been proposed that adipose tissue fibrosis in obese subjects caused by excess deposition of collagen, including type VI, leads to adipose tissue inflammation by triggering the infiltration of immune cells, such as macrophages, neutrophils, and lymphocytes (87, 182, 183). Pathological remodeling of ECM may also reduce the number of adipose tissue capillaries in obese and insulin-resistant subjects (184). Certain subtypes of collagen appear crucial for adipose tissue fibrosis-mediated inflammation and metabolic dysfunction in obesity (182, 183). Deletion of collagen VI in obese mice improves metabolic parameters and inflammatory profiles even though overall collagen content of the adipose tissue is not altered (182). However, it remains to be determined whether MMPs secreted from ATMs prevent collagen deposition linked to adipose tissue fibrosis.

Accumulation of ATMs is a key factor aggravating inflammatory responses in adipose tissues (2–4). However, recruited ATMs in obese adipose tissues may also play a protective role by enhancing angiogenesis in response to local hypoxia. Hypoxia is one of the most potent microenvironmental factors for macrophage recruitment into adipose tissues (185), and recruited macrophages are the predominant producers of pro-angiogenic factors in response to hypoxia (186). In mouse obese adipose tissues, ATM depletion decreases the expression level of platelet-derived growth factor (PDGF), an angiogenic factor that mediates endothelial cell tube formation and capillary maturation by pericyte recruitment (177). These results indicate that ATMs could be an important source of PDGF for angiogenesis. Furthermore, pro-inflammatory cytokines released from ATMs, such as TNFα, could act as angiogenic factors (187–189). Therefore, it has been speculated that ATMs may counteract hypoxia by promoting angiogenesis through production of angiogenic factors. However, recent elucidation of ATM-mediated angiogenesis in the obese adipose tissue still needs to be confirmed *in vivo*.

### Clearance of Dead Adipocytes and Macrophages

Another potential physiological role of ATMs in obesity is the clearance of dead or damaged adipocytes and fragmented cellular contents. In DIO mice, perilipin-negative adipocytes show features of necrosis-like death, such as plasma membrane rupture, dilated endoplasmic reticulum, and cell debris (80, 81). Most of the recruited ATMs in obese adipose tissues are present near perilipin-negative adipocytes, forming CLSs (80, 81). These CLS macrophages are multinucleated giant cells that surround residual adipocyte lipids, implying that CLS macrophages scavenge free lipids released from dead adipocytes to the interstitium (80). It was recently shown that depletion of mannose-binding lectin, which stimulates the phagocytic capacity of ATM, increases the numbers of CLSs in the adipose tissue of DIO mice, and decreases dead adipocyte clearance (190). Thus, it is likely that macrophage recruitment into the obese adipose tissue is involved in the clearance of dead adipocytes via phagocytosis. Furthermore, it has been suggested that ATMs could mediate the removal of extracellular lipids from adipose tissues (191). In both fasted and HFD-fed mice, ATMs absorb FFAs released from the adipocytes. This phenomenon could contribute to the prevention of lipotoxicity by buffering local extracellular FFA elevations in the adipose tissue. In obesity, however, lipid-loaded ATMs resembling foam cells have been suggested to have pro-inflammatory characteristics (192, 193) that contribute to insulin resistance. In both mice with adipocyte-targeted activation of caspase-8 and fat-transplanted mice, adipocyte death facilitates the recruitment of M1 and M2 macrophages (194, 195). It is possible that M1 macrophages phagocytize dead adipocytes, whereas M2 macrophages reconstruct ECM and resolve the activation of M1 macrophages after removal of dead adipocytes. These complementary functions of M1 and M2 macrophages may minimize tissue damage through tight regulation of phagocytosis and tissue repair. However, in obesity, the balance between M1 and M2 ATMs appears to be shifted by a greater increase in M1 macrophage number (2–4). Enhanced M1 polarization of ATMs in obese adipose tissues may interrupt the normal process of dead cell clearance, whereas further stimulating a pro-inflammatory response.

### Adaptive Thermogenesis and Adipose Tissue Macrophages

In response to cold exposure, β-adrenergic signaling modulates adaptive thermogenesis in BAT and SAT by promoting uncoupled respiration and thermogenic gene expression (40). Also, chronic cold exposure leads to the remodeling of BAT and SAT, resulting in a dramatic increase in the metabolic rate, mitochondrial biogenesis, and fatty acid oxidation (40). Emerging evidence suggests that M2 ATMs mediate thermogenic adipose tissue remodeling in response to cold exposure (196–198). Chawla’s group proposed that M2 ATMs induce thermogenic tissue remodeling by amplifying cold-induced β-adrenergic signaling via local catecholamine production (196, 198). In accordance with this notion, ATM depletion suppresses the cold-induced increase in thermogenic gene expression, lipolysis, and energy expenditure. In addition, blockage of M2 polarization by inhibition of IL-4 signaling diminishes adaptive thermogenesis and attenuates the generation of UCP-1-positive beige adipocytes in SAT. It has also been suggested that M2 ATMs promote the accumulation of PDGFRα-positive progenitor cells through osteopontin secretion and downstream β-adrenergic signaling (197). Recruited PDGFRα-positive progenitor cells can differentiate into UCP-1-positive adipocytes. Thus, it is likely that the interaction between M2 ATMs and PDGFRα-positive progenitor cells mediates the *de novo* differentiation of beige adipocytes in SAT during cold exposure. Together, these results indicate that ATM-associated adipose tissue remodeling is intimately involved in the modulation of adipose tissue function as well as adipose tissue growth and expansion in changing environments.

## Conclusion

We have focused on the physiological and pathological roles of the adipose tissue and its remodeling in regulating whole-body energy metabolism by sensing the nutritional status and crosstalk between adipocytes and stromal vascular cells. During adipose tissue expansion, numerous cellular responses are dynamically altered. Modulation of ECM remodeling and angiogenesis may promote differentiation of *de novo* adipocytes, preventing the formation of hypertrophic adipocytes by providing additional capacity to store extra lipids. Recruited ATMs probably act to remove dead adipocytes and repair damaged adipose tissue for healthy adipose tissue remodeling. Adipose tissue remodeling is modulated by the inflammatory responses of multiple immune cell types, including macrophages and lymphocytes. However, in obesity, chronic excessive energy storage in the adipose tissue initiates pathological remodeling, which triggers pro-inflammatory responses of immune cells. Furthermore, tissue remodeling, including angiogenesis and tissue repair for healthy expansion of the adipose tissue, is hindered by inflammatory and metabolic stress. Thus, resolving uncontrolled pro-inflammatory responses during adipose tissue remodeling is important for maintaining a metabolically healthy status. Adipose tissue remodeling is a complex but well-orchestrated mechanism modulated by multiple adipose tissue cell types that allow adaptation to external environmental changes. A deeper mechanistic understanding of adipose tissue remodeling could facilitate the development of therapeutic approaches against obesity-induced metabolic diseases.

## Author Contributions

SSC, JYH, IJH, JIK, and JBK contributed to the writing of the manuscript under JBK’s supervision.

## Conflict of Interest Statement

The authors declare that the research was conducted in the absence of any commercial or financial relationships that could be construed as a potential conflict of interest.
